# Joint optimization of system utility in UAV-enabled edge computing

**DOI:** 10.1371/journal.pone.0342583

**Published:** 2026-02-26

**Authors:** Huaiyu Zuo, Erqing Zhang, Yulong Tang, Mengxia Yin, Wu Dong

**Affiliations:** Beijing Institute of Graphic Communication, Beijing, China; Xidian University, CHINA

## Abstract

This paper addresses the joint optimization of system utility in Unmanned Aerial Vehicle (UAV)-enabled Mobile Edge Computing (MEC) networks. Unlike traditional approaches that optimize individual components, such as users, UAVs, or base stations, we propose a novel framework aimed at maximizing the overall system utility, which is defined as the difference between the total revenue of service providers (UAVs and base stations) and the total cost incurred by users. The proposed model incorporates realistic constraints, including limited computational resources and energy consumption, and formulates the problem as a Mixed-Integer Nonlinear Programming (MINLP) model. To solve this complex optimization problem, we develop an efficient algorithm that integrates the Block Successive Upper-Bound Minimization (BSUM) framework with heuristic methods, enabling the decomposition of the original problem into tractable subproblems that are solved iteratively. Simulation results demonstrate that the proposed approach significantly outperforms traditional heuristic algorithms in terms of system utility, while also exhibiting robust convergence and reliability across various network configurations. The results highlight the effectiveness of joint optimization in improving both the economic and operational efficiency of UAV-assisted MEC systems, providing a solid foundation for future research in network utility management.

## Introduction

The rapid advancement of information technology has led to the integration of Unmanned Aerial Vehicle (UAV) and Mobile Edge Computing (MEC) technologies, emerging as a promising solution to address the challenges faced by modern wireless networks. UAVs, with their inherent mobility and flexibility, complement MEC systems by providing on-demand computational and communication support in areas where traditional infrastructure may be limited. Specifically, UAVs can dynamically deploy computational resources, offload tasks, and enhance communication coverage in remote, disaster-stricken, or high-density areas, thereby improving the overall performance of MEC systems. MEC, by bringing computational and storage resources to the network edge, reduces latency and bandwidth requirements, enabling faster data processing and an enhanced user experience [1–4]. When combined, these technologies form a powerful synergy that addresses a wide range of challenges, including resource allocation, task offloading, and network scalability.

Despite the advantages of UAV-assisted MEC networks, optimizing their performance remains a complex and unresolved problem. Most existing studies focus on optimizing individual performance metrics, such as computation delay, energy consumption, or task offloading efficiency, often treating MEC and UAV technologies as separate entities. These studies tend to concentrate on optimizing UAV trajectories, task offloading strategies, or resource allocation independently, without accounting for the interdependencies between UAVs, base stations, and users. As a result, they adopt fragmented optimization approaches that fail to maximize the overall system utility. Furthermore, existing research often overlooks the critical balance between the revenues of service providers (UAVs and base stations) and the costs incurred by users, which is essential for ensuring the long-term sustainability of UAV-assisted MEC networks in real-world applications.

The core problem addressed in this paper is the joint optimization of system utility in UAV-assisted MEC networks. Specifically, the goal is to maximize overall system utility, defined as the difference between the total revenue generated by service providers (UAVs and base stations) and the costs incurred by users. This system utility is influenced by multiple factors, including task offloading decisions, resource allocation (both computational and communicative), and pricing strategies set by UAVs and base stations. The primary challenge lies in optimizing these interrelated variables under several practical constraints, such as limited computational resources, energy consumption, and network load. Unlike traditional approaches that independently optimize individual components (e.g., task offloading or resource allocation), this paper formulates a comprehensive, multi-dimensional optimization problem that jointly considers the interdependent decisions of service providers and users. The optimization problem is further complicated by its non-convexity and the complex interactions among decision variables, necessitating an integrated solution approach.

This study aims to address the gap in existing research by providing a unified framework for optimizing system utility in UAV-assisted MEC networks. The objective is to jointly optimize task offloading, resource allocation, and pricing strategies, with the goal of balancing the revenues of service providers and the costs incurred by users. The key contributions of this paper are as follows:

- Proposing a novel optimization framework that simultaneously considers the interdependencies between UAVs, base stations, and users to maximize system utility.
- Introducing a Mixed-Integer Nonlinear Programming (MINLP) model that incorporates a range of practical constraints, including limited resources, energy consumption, and network load.
- Developing an efficient algorithm that integrates the Block Successive Upper Bound Minimization (BSUM) framework with heuristic methods to effectively solve the non-convex optimization problem.
- Verifying the effectiveness of the proposed approach through simulation results, which demonstrate substantial improvements in system utility compared to traditional methods.

### Related work

In recent years, the rapid development of MEC and UAV technologies has led to a growing body of research focused on optimizing the collaborative operation of these two technologies. In UAV-assisted MEC networks, much of the research has concentrated on optimizing individual performance metrics, such as computation resource allocation, task offloading strategies, energy efficiency, and latency optimization. Current studies primarily focus on improving specific objectives, such as reducing computation delay, minimizing energy consumption, or enhancing task offloading efficiency, under predefined constraints. However, there remains a lack of research that comprehensively considers multiple performance metrics, particularly in the context of optimizing overall system utility.

Many studies focus on optimizing a single performance metric, such as computation delay, energy efficiency, or task offloading efficiency. For instance, [5] proposed a trajectory prediction-based edge offloading strategy to enhance the processing efficiency of computational tasks by optimizing the task offloading path. This method aims to reduce transmission latency through task offloading path optimization but does not consider the broader system utility, such as the balance between the utility of service providers (UAVs, base stations) and the costs incurred by users. Similarly, [2,6–10] applied game theory to examine how UAV-assisted MEC can improve system performance through computation offloading optimization, as well as optimize user scheduling, UAV management, and power allocation. While these game-theoretic approaches effectively optimize task offloading decisions, they focus mainly on optimizing individual objectives, such as energy efficiency or task offloading efficiency, without fully considering the global system utility. [11] proposed an efficient energy minimization scheme that optimizes UAV trajectory, resource allocation, and task offloading strategies, significantly reducing the network’s energy consumption. This approach transformed a non-convex optimization problem with multi-variable coupling into a more manageable form by introducing auxiliary variables, successfully achieving energy consumption optimization. However, the scheme primarily focuses on energy consumption. [6] optimized UAV trajectories and area division through a task offloading framework, reducing energy consumption during the task offloading process. The optimal transportation theory introduced in this study provided services to multiple users, successfully optimizing task offloading paths and energy efficiency. While it excels in terms of energy efficiency and offloading efficiency, it does not address the balance of benefits and costs between service providers and users, remaining limited to optimizing a single objective.

In UAV-assisted MEC systems, Deep Reinforcement Learning (DRL) [12–14] has been widely applied to optimize task offloading and trajectory design. [15] proposed a framework based on Double Deep Q-Learning (D-DQN) to optimize the 3D trajectory design and task offloading strategy of UAVs through online learning in dynamic environments. This method addresses dynamic optimization issues to some extent, enhancing task offloading and energy efficiency. However, it does not consider the trade-off between user costs and system revenues. [16] introduced a DRL-based approach aimed at optimizing UAV flight trajectories to maximize average secrecy rates in wireless security. While this study effectively improved system security, it focused solely on secrecy metrics and did not account for the balance among multiple performance metrics such as task offloading, energy efficiency, and latency. [17] proposed an iterative algorithm that jointly optimized UAV beamforming, CPU frequency, trajectory design, and user transmission power, successfully reducing the system’s energy consumption. Although this study provided valuable insights into resource allocation in UAV-assisted MEC systems, it remained focused on optimizing a single objective (energy efficiency).

In UAV-assisted MEC systems and Industrial Internet of Things (IIoT) environments [18,19], many studies have applied heuristic algorithms to optimize task offloading, resource allocation, and energy efficiency. [20] proposed a user satisfaction-oriented task offloading and UAV scheduling strategy to optimize task offloading and user satisfaction. This study employed a genetic algorithm (GA) and simulated annealing (SA) to combine task offloading decisions with UAV scheduling strategies, improving overall user satisfaction. However, it remains based on single-objective optimization and does not comprehensively balance multiple performance metrics. [21] focused on resource allocation in IIoT environments and proposed a learning-based resource allocation scheme using a Cooperative Particle Swarm Optimization (LCPSO) algorithm, successfully optimizing response time in forest fire monitoring. Although this approach improved response time, it did not sufficiently address the weight balancing problem across multiple performance metrics [22]. Furthermore, many existing studies, when faced with complex constraints, tend to rely on heuristic algorithms and convex optimization methods to solve problems. For example, an energy consumption minimization scheme based on a low-complexity resource allocation algorithm was proposed, which optimized user delay and energy efficiency, particularly in Heterogeneous Non-Orthogonal Multiple Access (HNOMA) networks. While this scheme significantly improved energy efficiency, it remained focused on energy efficiency alone, without a comprehensive improvement in system utility.

Previous research [23,24] applied the BSUM algorithm to optimize overall energy consumption and latency in task offloading within MEC systems. The novelty of this study lies in proposing a global system utility optimization scheme based on the BSUM framework. By comprehensively considering the differences between user costs and service provider (UAV and base station) revenues, a new multidimensional optimization framework is introduced. However, despite these advancements, most existing studies focus on optimizing individual performance metrics, such as computation delay, energy efficiency, or task offloading efficiency. These approaches typically overlook the balance between the revenues of service providers (UAVs and base stations) and the costs incurred by users. For instance, as shown in Table 1, studies like those of [23] primarily focus on optimizing specific metrics, such as task offloading and energy efficiency, but often fail to address the balance between provider revenues and user costs.

**Table 1:** Summary of key contributions and methodologies of selected studies on UAV-assisted MEC.

Research Focus	Methodology	Strengths	Weaknesses	Future Research Directions	Reference
Energy-efficient Resource Management in UAV-assisted MEC	BSUM algorithm to optimize energy consumption and UAV scheduling.	Reduces energy consumption while considering latency and load.	Assumes complete network state information, which may not be available.	Improve network state estimation in UAV MEC systems without centralized optimization.	[24]
Collaborative Computing Services at Ground, Air, and Space: An Optimization Approach	Optimizes offloading decisions between UAVs and satellites to minimize latency.	Integrates space, air, and ground systems for a unified MEC approach.	Limited focus on UAV collaboration and scalability in large networks.	Investigate scalability of UAV-enabled MEC systems and network expansion.	[25]
Reinforcement Learning Hyper-Heuristic Multi-Task Assignment of Multi-UAV	Game model with RL for task offloading and resource allocation in UAV networks.	Combines game theory and RL for dynamic resource allocation.	Does not address multi-service providers or large-scale UAV deployments.	Extend framework to include multiple service providers and UAV cooperation.	[26]
UAV Scheduling based on Heuristic Greedy Algorithm	Combines RL with a hyper-heuristic approach for task assignment.	Improves search efficiency and task assignment accuracy.	Heuristic methods may not guarantee the global optimal solution.	Develop methods to ensure global optimality in heuristic algorithms.	[27]
Multiple UAV Collaborative Task Assignment Using Reinforcement Learning	Grid-based heuristic scheduling to optimize UAV positioning and task assignment.	High accuracy and low computational complexity in scheduling.	Grid scheduling complexity may increase with more UAVs.	Investigate the impact of UAV count and flight dynamics on scheduling.	[28]
Service Satisfaction-Oriented Task Offloading and UAV Scheduling in UAV-Enabled MEC Networks	Proposes task priority models and a novel task offloading framework based on user satisfaction.	Maximizes user satisfaction by balancing task delay and energy saving.	The framework may need further refinement for large-scale deployments.	Investigate distributed scheduling methods for large UAV-enabled MEC systems.	[29]
Task Offloading and Trajectory Scheduling for UAV-Enabled MEC Networks	Jointly optimizes task offloading and UAV trajectory to minimize energy consumption.	Energy-efficient solution and load balancing among regions.	Assumes accurate SMD distribution, which might not be feasible in real-world scenarios.	Explore task offloading with unknown SMD locations in dynamic environments.	[30]
Learning-Based Resource Allocation Strategy for Industrial IoT in UAV-Enabled MEC Systems	Proposes an LCPSO algorithm for resource allocation in UAV-enabled MEC systems.	Considers priority constraints in IIoT for quicker response times.	Computationally complex due to the NP-hard nature of the problem.	Test LCPSO on larger-scale industrial IoT applications and networks.	[31]
Energy Consumption Minimization for Secure UAV-enabled MEC Networks Against Active Eavesdropping	Proposes energy minimization in secure UAV-enabled MEC systems with active eavesdropping.	Minimizes energy consumption while ensuring secure offloading.	Assumes perfect knowledge of network conditions, which may not be realistic.	Improve real-time security measures and adapt to evolving threats.	[32]

Furthermore, while some studies utilize the BSUM algorithm or other optimization techniques, they generally focus on single-objective optimization, such as minimizing energy consumption or enhancing task offloading efficiency, without considering the trade-off between service provider revenue and user costs. The current literature also lacks a unified, comprehensive framework capable of simultaneously addressing task offloading, resource allocation, and pricing strategies, while ensuring an optimal balance between service providers’ revenues and user costs.

## System model and problem formulation

**Fig 1:**
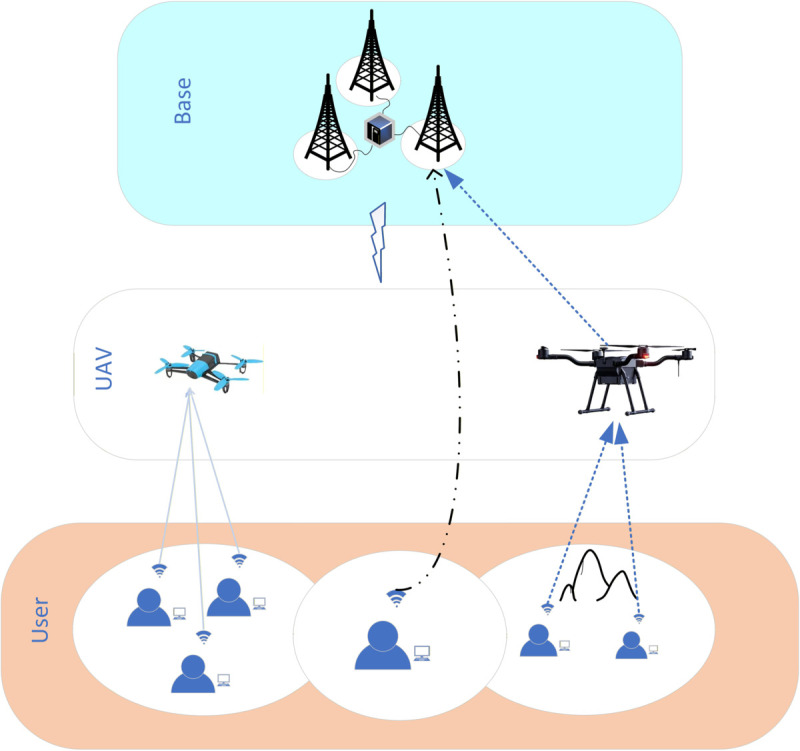
UAV-assisted MEC network model.

As shown in Fig 1, the system considers a UAV-assisted MEC network task offloading model. The model is divided into three layers: users, UAVs, and base stations, represented by sets *B*, *J*, and *I*, respectively. The UAV hovers above the users, providing edge computing offloading services. The UAVs’ positions are known to the users, and users prefer to offload tasks *D*_*i*_ to the UAV or base station that is closest to them. Each user has a specific task to offload. Users can choose from four task offloading methods: local offloading, offloading to the UAV for computation, offloading to the base station for computation, or offloading to the base station via UAV relay. Due to the limited computational resources, users can adopt any of these four offloading methods to handle tasks. Meanwhile, both the UAV and base station charge the users corresponding fees to ensure their own benefits.

### Base station layer

In the MEC network, ground base stations possess powerful computing capabilities and a continuous power supply. The set of users requiring task offloading is denoted as $i\in I=\{1,2,3,\ldots ,i_{\text{max}}\}$, where $i_{\text{max}}$ represents the maximum number of users in the set. Similarly, the sets of base stations and UAVs serving these users are represented as $b\in B=\{1,2,3,\ldots ,b_{\text{max}}\}$ and $j\in J=\{1,2,3,\ldots ,j_{\text{max}}\}$, where $b_{\text{max}}$ and $j_{\text{max}}$ denote the maximum number of base stations and UAVs in the respective sets.

For each base station *b*, user *i* can either directly offload tasks to the base station for computation or offload tasks to the base station via UAV *j* as a relay. The base station sets a price $M_{i}^{b}$ for the computational resources used by user *i*, and a price $M_{j}^{b}$ for the relay UAV *j*. The base station’s computational resources *F*^*b*^ are limited, and the resources allocated to user *i* are denoted by ${F\;}_{i}^{b}$. Let $\gamma_{i}^{b}\in \{0,1\}$ represent the user’s task offloading decision variable, where $\gamma_{i}^{b}=1$ indicates that the task is offloaded to the base station via a wireless link, and $\gamma_{i}^{b}=0$ otherwise. Similarly, $\gamma_{i}^{b,j}$ indicates whether the task of user *i* is offloaded to the base station through UAV *j* as a relay.

The offloading decisions can be mathematically expressed as follows:

$$\gamma_{i}^{b}=\left\{ \begin{array}{cc} 1, & \text{if the task }D\text{ of user }i\text{ is offloaded to base station }b\\ 0, & \text{otherwise} \end{array}\right.$$ (1)

$$\gamma_{i}^{b,j}=\left\{ \begin{array}{cc} 1, & \text{if the task }D\text{ of user }i\text{ is offloaded to base station }b\text{ via UAV }j\\ 0, & \text{otherwise} \end{array}\right.$$ (2)

The base station’s utility function is defined as $U_{b}=P_{b}-C_{b}$, where *P*_*b*_ represents the profit of the base station from providing computation services to users, and *C*_*b*_ is the cost of using UAVs. These can be expressed as:

$$P_{b}=\sum_{i\in I}\gamma_{i}^{b}M_{i}^{b}{F\;}_{i}^{b}$$ (3)

$$C_{b}=\sum_{i\in I}\sum_{j\in J}\gamma_{i}^{b,j}M_{i}^{b}D_{i}$$ (4)

### UAV layer design

In the MEC network, UAVs can serve either as base stations to receive and compute tasks offloaded by users, or as relay nodes to forward users’ tasks to base stations for computation. When a UAV functions as a base station, it sets a pricing $m_{i}^{j}$ for the computing resources allocated to user *i*. Similarly, the UAV’s computing resources *F*_*j*_ are limited. Let $\gamma_{i}^{j}$ denote the user task offloading decision variable, indicating whether user *i* offloads the task to UAV *j* for computation.

$$\gamma_{i}^{j}=\left\{ \begin{array}{cc} 1, & \text{if user }i\text{ offloads task }D\text{ to UAV }j\\ 0, & \text{otherwise} \end{array}\right.$$ (5)

When the UAV acts as a base station, it sets the pricing for the computing resources allocated to users, and similarly, the UAV’s computing resources are also constrained. The utility function of the UAV layer can be expressed as $U_{j}\;=\;P_{j}^{\text{compute}}+P_{j}^{\text{relay}}-C_{j}^{\text{compute}}-C_{j}^{\text{trans}}$, where $P_{j}^{\text{compute}}$ and $P_{j}^{\text{relay}}$ represent the UAV’s revenue when acting as a base station and a relay, respectively, while $C_{j}^{\text{compute}}$ and $C_{j}^{\text{trans}}$ represent the computational cost when the UAV acts as a base station and the communication cost for transmitting tasks as a relay, as shown below:

$$P_{j}^{\text{compute}}=\sum_{i\in I}\gamma_{i}^{j}m_{i}^{j}{f\;}_{i}^{j}$$ (6)

$$P_{j}^{\text{relay}}=\sum_{i\in I}\sum_{j\in J}\gamma_{i}^{b,j}M_{j}^{b}D_{i}$$ (7)

where ${f\;}_{i}^{j}$ represents the computing resources allocated to user *i* by UAV *j*, and

$$C_{j}^{\text{compute}}=\sum_{i\in I}\gamma_{i}^{j}\frac{\phi_{i}}{{f\;}_{i}^{j}}C_{i}$$ (8)

$$C_{j}^{\text{trans}}=\sum_{i\in I}\sum_{j\in J}\gamma_{i}^{b,j}P_{j}\frac{D_{i}}{{\text{rate}}_{i}^{b,j}}$$ (9)

where *P*_*j*_ is the transmission power of UAV *j*, and ${\text{rate}}_{i}^{b,j}$ is the transmission rate from UAV *j* to base station *b*. Let $\phi_{i}$ represent the number of CPU cycles required to complete the computation for user *i*.

### User layer design

In the MEC network, the utility of the user is determined by both the task offloading cost $C_{i}^{\text{pay}}$ and the processing cost $C_{i}^{\text{compute}}$, which includes computation delay and communication cost during task transmission. Let $\gamma_{i}^{l}\in \{0,1\}$ represent the user’s decision variable indicating whether user *i* performs local computation for the task.

$$\gamma_{i}^{l}=\left\{ \begin{array}{cc} 1, & \text{if user }i\text{ performs local computation for task }D\\ 0, & \text{otherwise} \end{array}\right.$$ (10)

The task offloading cost $C_{i}^{\text{pay}}$ is defined as:

$$C_{i}^{\text{pay}}=\left\{ \begin{array}{cc} 0, & \gamma_{i}^{l}\geq 0\\ M_{i}^{b}{F\;}_{i}^{b}, & \gamma_{i}^{b}\geq 0\\ m_{i}^{j}{f\;}_{i}^{j}, & \gamma_{i}^{j}\geq 0\\ M_{j}^{b}{F\;}_{i}^{b}, & \gamma_{i}^{j,b}\geq 0 \end{array}\right.$$ (11)

The computation cost $C_{i}^{\text{compute}}$ is expressed as:

$$C_{i}^{\text{compute}}=\left\{ \begin{array}{cc} \frac{\phi_{i}}{{f\;}_{i}}+\frac{\phi_{i}}{{f\;}_{i}}C_{i}, & \gamma_{i}^{l}\geq 0\\ \frac{D_{i}}{{\text{rate}}_{i}}+\frac{\phi_{i}}{{F\;}_{i}}+\frac{P_{i}D_{i}}{{\text{rate}}_{i}}, & \gamma_{i}^{b}\geq 0\\ \frac{D_{i}}{{\text{rate}}_{i}}+\frac{\phi_{i}}{{f\;}_{i}}+\frac{P_{i}D_{i}}{{\text{rate}}_{i}}, & \gamma_{i}^{j}\geq 0\\ \frac{D_{i}}{{\text{rate}}_{i}}+\frac{\phi_{i}}{{F\;}_{i}}+\frac{D_{i}}{{\text{rate}}_{i}^{b,j}}+\frac{P_{i}D_{i}}{{\text{rate}}_{i}^{b}}, & \gamma_{i}^{j,b}\geq 0 \end{array}\right.$$ (12)

where *P*_*i*_ is the transmission power of user *i*, and $\gamma_{i}^{l},\gamma_{i}^{b},\gamma_{i}^{j},\gamma_{i}^{j,b}$ represent the user’s offloading decisions. Based on these, the utility of the user layer can be expressed as $U_{i}=-C_{i}^{\text{pay}}-C_{i}^{\text{compute}}$ (Table 2).

**Table 2:** Summary of assumptions, variables, and system parameters.

Symbol	Description	Assumptions/Constraints
*I*	Set of users	$I=\{1,2,\ldots ,i_{\text{max}}\}$
*J*	Set of UAVs	$J=\{1,2,\ldots ,j_{\text{max}}\}$
*B*	Set of base stations	$B=\{1,2,\ldots ,b_{\text{max}}\}$
$F_{i}^{b}$	Computation resources allocated by base station *b* to user *i*	$F_{i}^{b}\leq F^{b}$
$M_{i}^{b}$	Pricing set by base station *b* for user *i*	$M_{i}^{b}\in [\text{min},\text{max}]$
$\gamma_{i}^{b}$	Offloading decision variable for base station *b* and user *i*	$\gamma_{i}^{b}\in \{0,1\}$
$\gamma_{i}^{j}$	Offloading decision variable for UAV *j* and user *i*	$\gamma_{i}^{j}\in \{0,1\}$
*P*_*i*_	Transmission power of user *i*	Given in system parameters
*C*_*i*_	Energy consumption per CPU cycle for user *i*	Given in system parameters

### System utility and optimization problem

In the user offloading system designed for our MEC network, the goal is to maximize the overall system utility while ensuring that constraints, such as UAV and base station computing resources and offloading decisions, are satisfied. In the proposed system model, the interactions between users, UAVs, and base stations are critical for optimizing overall system utility. Users offload their computational tasks to the nearest UAV or base station, depending on resource availability and pricing strategies set by the UAV and base station. UAVs, acting as either relays or computation units, interact with both users and base stations to maximize the overall system utility by optimizing the offloading decisions, resource allocation, and pricing schemes. Base stations, with powerful computing resources, set prices and allocate resources to users either directly or via UAVs. Thus, the system-level utility depends on the joint decisions made by users, UAVs, and base stations, making their interactions a key part of the optimization problem.

The overall system utility is defined as the difference between the total revenue generated by the service providers (UAVs and base stations) and the costs incurred by the users. Therefore, the system utility is influenced by task offloading decisions, resource allocation (computation and communication), and pricing strategies. The utility function for the overall system is defined as *U* = *U*_*b*_  +  *U*_*j*_ − *U*_*i*_, where *U*_*b*_ represents the utility of the base station, *U*_*j*_ represents the utility of the UAV, and *U*_*i*_ represents the utility of the user.

To facilitate the optimization process and make the problem compatible with standard optimization techniques, we transform the objective from maximizing *U* to minimizing −*U*. This transformation is commonly used in optimization, as most solvers are designed to minimize objective functions. Maximizing *U* is equivalent to minimizing −*U*, as the optimal solution remains unchanged, and the problem is computationally more convenient when expressed as a minimization problem.

Thus, the objective becomes minimizing −*U*, which is mathematically equivalent to maximizing *U*. Our optimization problem can be reformulated as:

$$T(𝐌,𝐉,\gamma )=U_{i}-U_{b}-U_{j}$$ (13)

This transformation ensures that the optimization problem remains consistent with the goal of maximizing system utility, while leveraging optimization techniques that are optimized for minimization problems.

Finally, we can express the formula for the overall system utility as:

minimize T(𝐌,𝐉,γ) (14)

The bounds for the pricing and offloading parameters, such as $M_{i}^{b}$, $M_{j}^{b}$, and $m_{i}^{j}$, are determined based on practical considerations and typical network operations. The minimum values are constrained by the operational costs of maintaining the UAVs and base stations, ensuring that these services remain economically viable. On the other hand, the maximum values are constrained by market conditions and competitive pricing, ensuring that pricing does not become prohibitively high for users, which could lead to inefficiencies in offloading decisions. The offloading parameters are similarly bounded by the available computational and communication resources of the network, ensuring that offloading decisions remain feasible within the system’s capabilities.


$$M_{i}^{b}\in [\text{min}(M_{i}^{b}),\text{max}(M_{i}^{b})],\;M_{j}^{b}\in [\text{min}(M_{j}^{b}),\text{max}(M_{j}^{b})],$$

$$m_{i}^{j}\in [\text{min}(m_{i}^{j}),\text{max}(m_{i}^{j})],\;\forall i\in I,\forall j\in J$$ (15)

These bounds ensure that the system operates within realistic parameters while maintaining both efficiency and feasibility.

Eq (17) represents the constraint on the computing resources allocated to all users by the base station and UAV. Eq (18) defines a range constraint on the pricing set by the base station and UAV for all users. Eq (19) indicates that each user can only choose one offloading method, and the decision is binary. Due to the coupling, non-linearity, binary constraints, and the non-convex structure of the decision variables in the objective function, convex optimization techniques cannot be directly applied to solve the problem.

To effectively solve the non-convex offloading optimization problem, we adopt the Block Successive Upper Bound Minimization (BSUM) method. BSUM decomposes the problem into several subproblems and constructs a local convex approximation for each subproblem, effectively avoiding the convergence issues that traditional methods, such as the Alternating Direction Method of Multipliers (ADMM), may encounter when dealing with non-convex problems. Additionally, BSUM offers lower computational complexity, making it suitable for solving large-scale, multi-task decision-variable optimization problems. In our case, BSUM demonstrates superior performance, particularly when handling complex non-convex optimization problems that involve both discrete and continuous decision variables.

In the BSUM framework, the upper bound of the objective function is minimized iteratively over the variables 𝐌, 𝐉, and γ. To apply the BSUM framework, the binary decision variable constraints in Eq (19) are relaxed into continuous constraints, i.e., $\gamma_{i}^{l}\in [0,1]$, $\gamma_{i}^{b}\in [0,1]$, $\gamma_{i}^{j}\in [0,1]$, and $\gamma_{i}^{j,b}\in [0,1]$. This relaxation is a standard approach in non-convex optimization that simplifies the problem, enabling the use of convex optimization techniques more effectively. By treating the binary variables as continuous, we can solve the problem using standard optimization methods. However, this relaxation introduces potential errors, as the continuous solutions may not directly satisfy the original binary constraints. To mitigate this, a post-processing step is applied in which the continuous values are rounded to the nearest binary values (0 or 1), ensuring that the final solution adheres to the original binary constraints.

These methods guarantee that the solution complies with the original problem’s binary constraints. Although the relaxation introduces some error, it does not significantly affect the optimality of the solution, as the resulting binary solution is close to the continuous optimal solution. This relaxation technique provides a more tractable approach to solving complex non-convex problems, while still maintaining feasibility and optimality with respect to the original binary constraints. We can now introduce the feasible set of 𝐌, 𝐉, and γ.

minimize T(𝐌,𝐉,γ) (16)

$$ℳ≜\{M_{i}^{b}\in [\min M_{i}^{b},\max M_{i}^{b}],\;M_{j}^{b}\in [\min M_{j}^{b},\max M_{j}^{b}],$$

$$m_{i}^{j}\in [\min m_{i}^{j},\max m_{i}^{j}],\;\forall i\in I,\forall j\in J\}$$ (17)

$$𝐉≜\left\{𝐉:\sum_{i\in I}{F\;}_{i}^{b}\leq F_{b},\;\sum_{j\in J}{f\;}_{i}^{j}\leq F_{j},\;{F\;}_{i}^{b}\leq D_{i},\;{f\;}_{i}^{j}\leq D_{i},\;\forall i\in I,\;\forall j\in J\right\}$$ (18)

$$\gamma ≜\{\gamma_{i}^{l}\in \{0,1\},\;\gamma_{i}^{b}\in \{0,1\},\;\gamma_{i}^{j}\in \{0,1\},\;\gamma_{i}^{j,b}\in \{0,1\},$$ (19)

$$\gamma_{i}^{l}+\gamma_{i}^{b}+\gamma_{i}^{j}+\gamma_{i}^{j,b}=1,\;\forall i\in I,\;\forall j\in J\}$$

Finally, we define the closest upper bound function *T*_*n*_ for the objective function in Eq (13). For each iteration *t*, n ∈ N, where *N* is the index set. To ensure that the upper bound function is more beneficial for our optimization, we apply a quadratic penalty to the objective function in Eq (13), as follows:

$$T(M_{n};M^{t},J^{t},\gamma^{t})=T(M_{n};M^{\left(t-1\right)},J^{\left(t-1\right)},\gamma^{\left(t-1\right)})+\frac{\theta_{n}}{2}\|M_{n}-M^{\left(t-1\right)}{\|}^{2}$$ (20)

where $\theta_{n}$ is the penalty parameter, which can also be applied to other variables. Additionally, in each iteration *t*, the upper bound function is minimized with respect to $M^{t},J^{t},\gamma^{t}$ using the values $M^{\left(t-1\right)},J^{\left(t-1\right)},\gamma^{\left(t-1\right)}$, which are the solutions from the previous iteration. The solution at iteration *t* + 1 can be updated by solving the following optimization problem:

$$M_{n}^{\left(t+1\right)}\in \arg\min_{M_{n}}T_{n}(M_{n};M^{t},J^{t},\gamma^{t})$$ (21)

$$J_{n}^{\left(t+1\right)}\in \arg\min_{J_{n}}T_{n}(J_{n};M^{t},J^{t},\gamma^{t})$$ (22)

$$\gamma_{n}^{\left(t+1\right)}\in \arg\min_{\gamma_{n}}T_{n}(\gamma_{n};M^{t},J^{t},\gamma^{t})$$ (23)

The iterative procedure in the BSUM framework updates the decision variables (*M*, *J*, and γ) in each iteration by minimizing a surrogate objective function for each respective variable. Each subproblem is solved independently, and the solution is updated iteratively until convergence. The convergence of this iterative process is guaranteed under the assumptions of the BSUM framework, which ensures convergence to a stationary point due to the block structure and the use of upper-bound minimization. However, since the problem is non-convex, convergence to the global optimum is not guaranteed. Each iteration involves solving three subproblems corresponding to *M*, *J*, and γ, with the complexity of each subproblem depending on the number of users, UAVs, and base stations. Specifically, solving each subproblem requires linear or quadratic optimization, so the overall complexity per iteration is O(n ⋅ m), where *n* represents the number of decision variables and *m* is the number of iterations required for convergence.

In the BSUM framework, we must also solve subproblems for three sets of variables. Since task offloading, pricing, and resource allocation involve multiple decision variables and complex constraints, and the optimization objectives are often nonlinear, traditional exact optimization methods (such as linear programming and integer programming) are frequently unable to find the global optimal solution within a reasonable time for large-scale problems, due to the vast solution space. Heuristic algorithms, which do not rely on the continuity or differentiability of the problem, are more suitable for handling complex combinatorial optimization problems. For the optimization of pricing variables, resource allocation, and offloading strategies, the interdependencies between variables and the complex constraints make exact solutions difficult. Heuristic algorithms can flexibly adapt to these changes, handle discrete decision spaces and complex constraints, and thus provide effective solutions. Therefore, the use of heuristic algorithms, such as GAs and SA, offers an effective solution.

**Algorithm 1 Joint optimization of system utility based on the BSUM framework.**

1: **Initialization:** Set *t* = 0, ϵ>0, and find the initial feasible solution.

2: **repeat** 3:   Select the index set *N*.

4:   Set $M_{n}^{t+1}\in \arg\min_{M_{n}}T_{n}(M_{n};M^{t},J^{t},\gamma^{t})$.

5:   Set $M_{m}^{t+1}=M_{m}^{t},\forall m\in N$.

6:   **Interface with GA:** Pass the continuous decision variables ($M_{n}^{t+1}$, $J_{n}^{t+1}$, $\gamma_{n}^{t+1}$) to the GA to optimize the discrete decision variables.

7:   **Update with GA output:** Receive the optimized discrete variables from GA and update $M_{n}^{t+1}$, $J_{n}^{t+1}$, $\gamma_{n}^{t+1}$.

8:   **Interface with SA:** Pass the updated continuous variables to the SA algorithm for further refinement of the continuous decision variables.

9:   **Update with SA output:** Receive the refined continuous variables from SA and update $M_{n}^{t+1}$, $J_{n}^{t+1}$, $\gamma_{n}^{t+1}$.

10:   Set *t* = *t* + 1.

11: **unit** $\left\|\frac{T_{n}^{t+1}-T_{n}^{t}}{T_{n}^{t}}\right\|\leq \epsilon$

12: **Final Solution:** Set $M_{n}^{t+1}$, $J_{n}^{t+1}$, and $\gamma_{n}^{t+1}$ as the required solution.

Optimization of Pricing Variables: GA. The optimization of pricing variables involves the pricing $M_{i}^{b}$ set by the base station for users, the pricing $M_{j}^{b}$ set by the base station for UAVs, and the pricing $m_{i}^{j}$ set by the UAV for users. These pricing variables need to be optimized under multiple constraints, with the goal of maximizing system benefits and reducing costs.

$$M_{n}^{\left(t+1\right)}\in \arg\min_{M_{n}}T_{n}(M_{n};M^{t},J^{t},\gamma^{t})$$ (24)

The Genetic Algorithm (GA) is used to optimize the pricing variables, including those set by base stations and UAVs. The population size is set to 200, striking a balance between solution diversity and computational efficiency. Tournament selection is employed with a tournament size of 4, which helps maintain diversity and avoid premature convergence. The GA terminates either after 50 generations or when the fitness improvement is smaller than a threshold ($\epsilon =10^{-6}$).

The crossover rate is set to 0.9, and the mutation rate is set to 0.25, based on preliminary testing. A sensitivity analysis showed that the algorithm remains stable across a range of parameters, demonstrating robustness. The GA evaluates and selects solutions, iterating through crossover and mutation steps to improve the pricing strategy and maximize overall system utility.

**Algorithm 2 Optimizing pricing variables using GA.**

1: Randomly initialize the population $\left(M_{i}^{b},M_{j}^{b},m_{i}^{j}\right)$.

2: Define the fitness function based on the system objective.

3: Select the index set *N*.

4: Select individuals based on their fitness.

5: Perform crossover and mutation operations to generate new individuals.

6: Update the population with the newly generated individuals.

7: Repeat the steps until convergence (maximum generations or fitness threshold is reached).

8: **repeat**

9:   $\left\|\frac{T_{n}^{t+1}-T_{n}^{t}}{T_{n}^{t}}\right\|\leq \epsilon$

10: **until**

11: Output the optimized pricing variables $\left(M_{i}^{b},M_{j}^{b},m_{i}^{j}\right)$.

**Optimization of Resource Allocation Variables: SA Algorithm.** The optimization of resource allocation variables involves the distribution of computing resources ${F\;}_{i}^{b}$ and ${F\;}_{i}^{j}$ by the base station and UAV. The optimization objective is to maximize the utilization of computing resources while ensuring that the resource allocation meets the system’s demands and constraints.

$$J_{n}^{\left(t+1\right)}\in \arg\min_{J_{n}}T_{n}(J_{n};M^{t},J^{t},\gamma^{t})$$ (25)

The SA algorithm is used to optimize the resource allocation variables. The SA algorithm perturbs the current solution randomly and uses the Metropolis criterion to decide whether to accept the new solution, thereby avoiding getting stuck in local optima.

**Algorithm 3 Optimizing resource allocation variables using SA.**

1: Initialize the resource allocation variables $\left({F\;}_{i}^{b},{F\;}_{i}^{j}\right)$.

2: Define the objective function for resource allocation.

3: Set the initial temperature and cooling coefficient.

4: **while** the temperature is greater than the final temperature **do**

5:   Generate a new solution by perturbing the current solution.

6:   Calculate the objective function value of the new solution.

7:   Accept the new solution based on the Metropolis criterion, with the acceptance probability depending on the temperature.

8:   Lower the temperature.

9: **end while**

10: Output the optimized resource allocation variables $\left({F\;}_{i}^{b},{F\;}_{i}^{j}\right)$.

The optimization of offloading strategy variables involves the user’s task offloading decisions $\gamma_{i}^{l}\in [0,1],\gamma_{i}^{b}\in [0,1],\gamma_{i}^{j}\in [0,1],\gamma_{i}^{j,b}\in [0,1]$. These decisions need to be optimized based on the user’s computational requirements, network conditions, and resource availability to maximize the system’s utility.

$$\gamma_{n}^{\left(t+1\right)}\in \arg\min_{\gamma_{n}}T_{n}(\gamma_{n};M^{t},J^{t},\gamma^{t})$$ (26)

The offloading strategy variables are also optimized using the SA algorithm. The algorithm steps are outlined in Algorithm 3, where the offloading strategy is randomly adjusted, and the new offloading scheme is evaluated based on the objective function. This process gradually converges to the optimal solution.

The solution methods for these three optimization problems involve the use of GAs and SA algorithms to optimize the pricing variables, resource allocation variables, and offloading strategy variables, respectively. The GA optimizes pricing and resource allocation through a global search, while the SA algorithm optimizes the offloading strategy by gradually cooling down, ensuring optimal performance of the entire system under complex constraints.

## Simulation and result analysis

To evaluate the proposed solution, the simulation environment consists of an MEC network with two base stations, six UAVs, and eight users. The locations of the base stations, UAVs, and users are distributed in a Cartesian coordinate system to facilitate task offloading. The remaining simulation parameters, including transmission power, resource allocation, and task offloading settings, are shown in Table 3. Specifically, the transmission power values for users and UAVs were chosen based on typical values used in UAV-assisted MEC systems from previous studies. The resource allocation values were adjusted through experimentation to balance system efficiency and user fairness.

**Table 3:** Other simulation parameter settings.

Parameter	Symbolic Representation	Value
*P*_*i*_	Transmission power of each user	8 W
*P*_*j*_	Transmission power of each UAV	5 W
*C*_*i*_	Energy consumption per CPU cycle	8 J
$\theta_{n}$	Weight of adjustment factor	0.6
popsize	Population size in the GA	200
maxgen	Maximum number of iterations in the GA	50
pcrossover	Crossover probability in the GA	0.9
pmutation	Mutation probability in the GA	0.25
initial_temp	Initial temperature in SA algorithm	300
final_temp	Termination temperature in SA algorithm	1e-3
alpha	Cooling rate in SA algorithm	0.97

**Fig 2:**
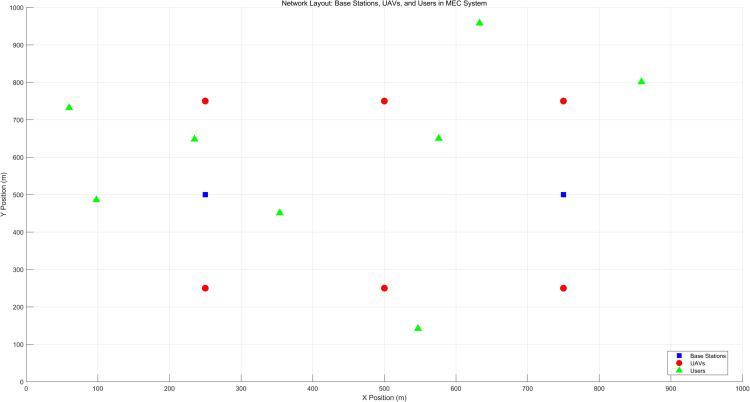
Location distribution in simulation.

A schematic illustrating the spatial distribution of base stations, UAVs, and users in the simulation setup is shown in Fig 2. The figure clearly depicts the placement of the nodes (base stations, UAVs, and users) in the Cartesian coordinate system. This layout allows for the simulation of offloading tasks, where users connect to either the base stations or UAVs for task offloading. The communication topology between the nodes is defined by wireless links, with users offloading tasks to the nearest base station or UAV based on distance and resource availability.

**Fig 3:**
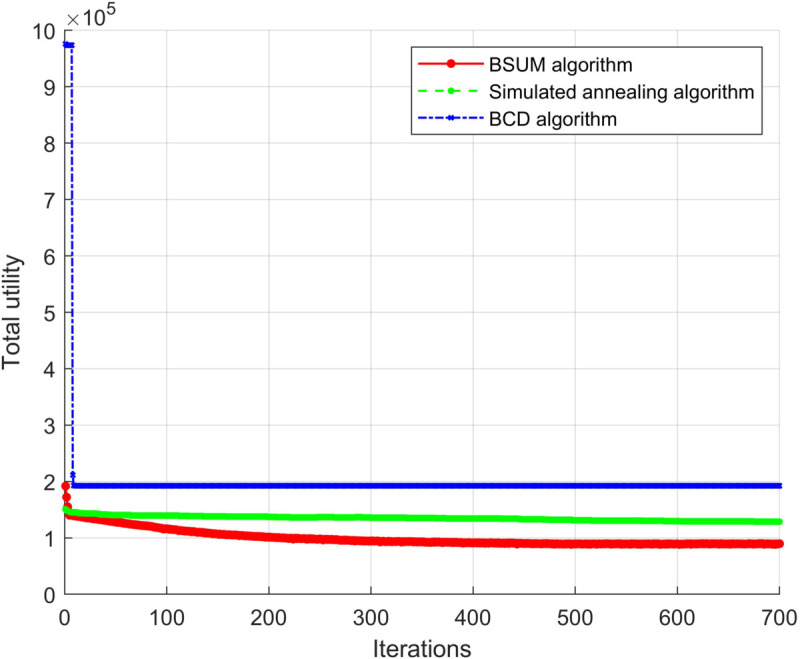
Algorithm convergence and comparison plot.

We present the convergence of different algorithms during the iteration process in Fig 3. As shown in the tables and figures, the BSUM algorithm demonstrates a clear advantage over the Block Coordinate Descent (BCD) and SA algorithms. For instance, with 8 users, BSUM achieves a system utility of 89,217, while BCD and SA achieve 575,832 and 122,182, respectively. This results in a percentage improvement of approximately 46.5% for BSUM over BCD and 26.9% over SA. As the number of users increases, the performance gap becomes even more pronounced, with BSUM outperforming BCD by more than 40% and SA by over 60% for 24 users. These results clearly demonstrate the superior efficiency and utility of the BSUM algorithm across different network configurations.

The BSUM algorithm not only converges in fewer iterations but also achieves a significantly higher total utility at the end, indicating that it performs more efficiently in handling complex optimization problems.

**Fig 4:**
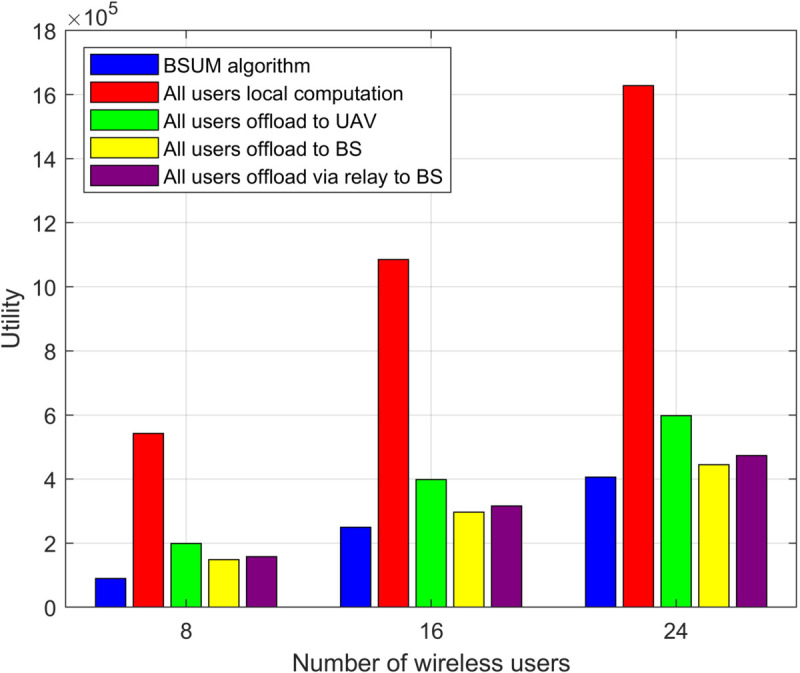
Utility of offloading destinations with different numbers of users.

Fig 4 shows the comparison of system utility under different numbers of users (8, 16, and 24 users) for various offloading strategies. The overall utility using the BSUM algorithm is significantly higher than with other offloading methods. Particularly when the number of users is 8 and 24, the BSUM algorithm demonstrates a considerable improvement in utility. For the “all users local computation” scenario, the system utility is relatively low, indicating that local computation does not effectively enhance system performance. Furthermore, offloading tasks through UAVs or base stations provides some utility improvement, but it is not as effective as the optimization achieved by the BSUM algorithm.

**Fig 5:**
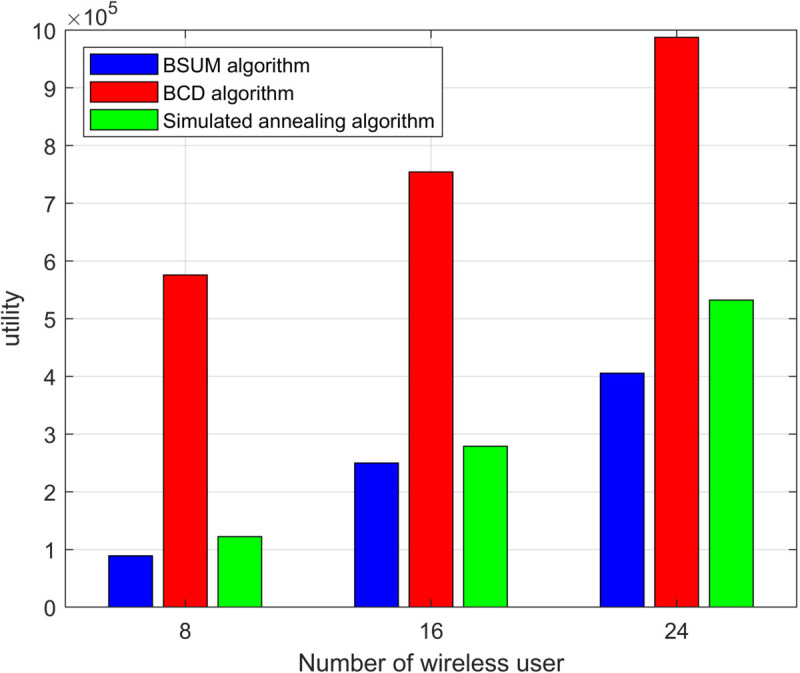
Utility of the algorithm under different numbers of users.

Fig 5 further compares the total utility of the BSUM algorithm with the other two optimization algorithms (BCD and SA) under different numbers of users. It is evident that the BSUM algorithm outperforms both the BCD and SA algorithms in all user scenarios. Particularly when the number of users is 24, the BSUM algorithm demonstrates a clear advantage in terms of system utility, outperforming the other two algorithms. This indicates that the BSUM algorithm can effectively improve resource utilization efficiency and overall system performance in large-scale user environments and complex network settings.

**Fig 6:**
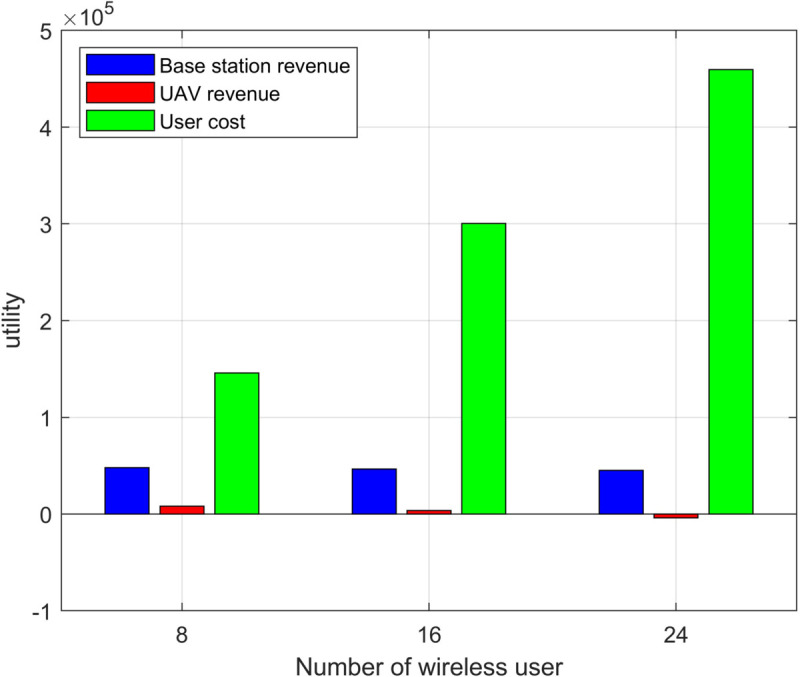
Utility of each component of the system under different numbers of users.

Fig 6 shows the changes in base station revenue, UAV revenue, and user costs under different numbers of users. In all cases, the revenue of the base stations and UAVs is much lower than the costs of the users, especially as the number of users increases, leading to a significant rise in user costs.

**Fig 7:**
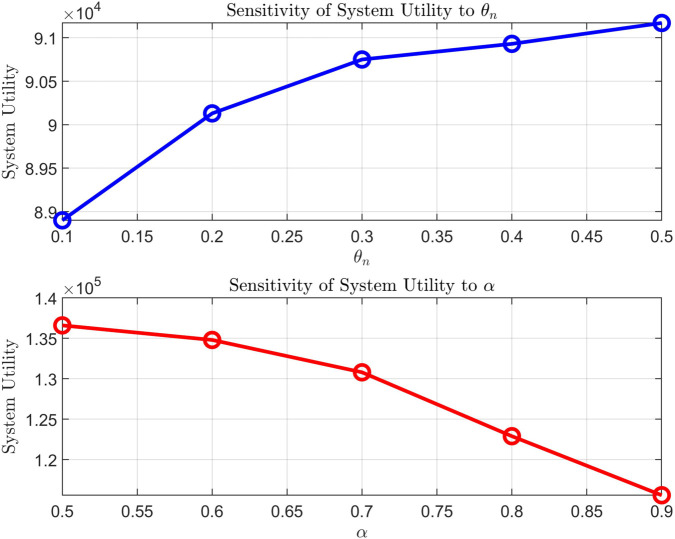
Sensitivity analysis of system utility to the variation of $\theta_{n}$ and α.

To further investigate the effect of key parameters on system performance, Fig 7 presents the sensitivity analysis of system utility to the variation of $\theta_{n}$ and α. The parameter $\theta_{n}$ is a penalty factor that controls the trade-off between maximizing system utility and ensuring that constraints are met, such as resource limits and cost balances. As $\theta_{n}$ increases, system utility rises, reflecting that a stronger penalty on constraint violations leads to a more balanced and efficient system, optimizing the trade-off between revenue and user costs. On the other hand, α is related to the resource allocation efficiency or the system’s pricing strategy. As α increases, system utility decreases, indicating that higher values of α may increase costs or reduce resource allocation efficiency, ultimately affecting the system’s overall utility.

## Conclusion

This paper addresses the system utility optimization problem in UAV-assisted MEC networks by proposing a new optimization framework. The goal is to maximize overall system utility, defined as the difference between the revenues of UAVs and base stations and the costs incurred by users. Unlike traditional single-objective optimization approaches, this study considers multiple constraints, including computational resources and energy consumption. The problem is formulated as a Mixed-Integer Nonlinear Programming (MINLP) problem, and the solution is obtained by combining the Block Successive Upper Bound Minimization (BSUM) framework with heuristic algorithms.

Simulation results show that the proposed BSUM algorithm performs effectively across various scenarios, significantly improving system utility. As the number of users increases, the system optimizes resource allocation and task offloading strategies more efficiently, although resource usage also rises with the user count. The analysis further compares the BSUM algorithm with traditional heuristics, highlighting its advantages in terms of resource consumption and utility under varying user loads.

However, the study has limitations. The simulations were conducted in a small-scale setup with idealized channel conditions, and real-world systems may involve more complex interactions in larger, dynamic networks. Additionally, the absence of mobility modeling for UAVs and users means that the impact of mobility on network performance was not considered. These factors may affect the system’s performance in practical deployments.

Despite these limitations, the proposed framework demonstrates strong convergence and robustness in controlled environments, indicating its scalability potential for larger networks. Future research will focus on incorporating mobility effects, realistic channel models, and testing the framework in larger, more dynamic network setups. Furthermore, we aim to explore energy-efficient task offloading strategies and real-time adaptability to address challenges such as network congestion and environmental factors in real-world applications.

## Simulation setup and computational environment

All simulations were conducted using MATLAB R2019b. The experiments were executed on a laptop with the following hardware configuration:

- CPU: AMD Ryzen 7 7735H with Radeon Graphics, 8 cores, 16 logical processors, clock speed of 3.2 GHz.
- GPU: NVIDIA GeForce RTX 4060 Laptop GPU with 4 GB memory, core frequency of 480 MHz, and memory frequency of 6000 MHz.

The simulations were run for an average duration of five hours depending on the specific scenario, with the results obtained reflecting the performance of the proposed algorithm under typical system conditions. The choice of MATLAB and this hardware setup ensures the computational efficiency and scalability of the approach for the network sizes considered in this study.

These details are provided to enable reproducibility of our results and fair performance comparison by other researchers using similar setups.

## Supporting information

S1 File Code. This file contains all the code for the experiments in the manuscript.
(ZIP)
